# The Use of a Smartphone to Assess the Two-Minute Step Test: Validity of Machine Learning Compared to Analytical Data Processing

**DOI:** 10.3390/s26051520

**Published:** 2026-02-28

**Authors:** Gustavo de Oliveira Hoffmann, Guilerme Parra Martini, John G. Buckley, Andre Luiz Felix Rodacki

**Affiliations:** 1 Center for Motor Behaviour Studies, Department of Physical Education, Paraná Federal University, Centro Politécnico, Rua Herculano F. dos Santos, 100–Jardim das Américas, Curitiba 81530-000, PR, Brazil; gustavohoffmann@ufpr.br (G.d.O.H.); guilhermemartini14@gmail.com (G.P.M.); rodacki@ufpr.br (A.L.F.R.); 2 School of Engineering, University of Bradford, Bradford BD7 1DP, UK

**Keywords:** two-minute step test, smartphone sensors, functionality, machine learning

## Abstract

**Highlights:**

**What are the main findings?**

- A smartphone held to the thigh provided valid estimates of several parameters of the 2MST.
- A machine learning data processing approach provided peak thigh angular velocity with better agreement to the ground truth measure than an analytical data processing approach.

**What are the implications of the main findings?**

- Holding a smartphone to the thigh provides a simple, easy, and low-cost way to measure 2MST parameters.
- The better agreement for machine learning-determined thigh peak angular velocity suggests that such an approach should be considered when using a smartphone to determine 2MST parameters.

**Abstract:**

The 2-Minute Step Test (2MST) is commonly scored by step count, which overlooks how the task is performed. This study tested whether a smartphone held to the thigh can be used to quantify thigh kinematics to determine 2MST outcome parameters, and whether a machine learning (ML) data analysis approach of the smartphone signal yields better agreement with motion capture (ground truth) compared to a more typical analytical data analysis approach (AA). Eighty-four healthy adults completed the 2MST while holding a smartphone against the right thigh. A thigh angular velocity ‘ground truth’ reference was obtained by simultaneous recording via motion capture (Vicon). Smartphone signals were resampled and processed using analytical (i.e., adaptive Butterworth filtering) and machine-learning data processing approaches (i.e., a stacked regression model trained to identify peak angular velocities). Step cycles and cycle duration were identical across equipment modalities and data analysis pipelines (mean 143 ± 18 cycles; 0.84 ± 0.11 s). However, the mean and variability of peak thigh angular velocity differed across the different modalities/pipelines (motion capture: 303 ± 39°·s^−1^; AA: 280 ± 47°·s^−1^; ML: 304 ± 37°·s^−1^). Bland–Altman agreement, compared to the ground truth measure, showed larger bias and limits of agreement for AA (bias 25.5°·s^−1^; −49.8–100.8) compared to ML (bias 1.0°·s^−1^; −15.4–17.5). These findings support using a smartphone held to the thigh to assess how the 2MST is performed, including providing the number and timing of steps completed and the average and variability in thigh angular velocity across cycles. Findings also suggest that a machine learning data analysis approach provides thigh angular velocity measures that are nearly identical to motion capture techniques, whereas a typical analytical data analysis approach has errors of around 8%.

## 1. Introduction

The assessment of aerobic endurance and lower-body functional fitness is often used to evaluate motor performance across clinical and community-dwelling populations, particularly in older adults and individuals with movement impairments. Among various field-based assessments, the 2-Minute Step Test (2MST) has emerged as a practical and straightforward tool for gauging lower-limb coordination, endurance, and postural control [1,2]. It has been commonly used in clinical and community settings, especially in older adults and individuals with limited mobility. During the test, participants are instructed to march in place as fast as possible for 2 minutes, raising each knee to a predetermined height—set at midway between the patella and the iliac crest. The number of times the right knee reaches the target height is counted and recorded as the test score.

Although the 2MST serves as a practical alternative to the 6 min walk test when space is limited, its associations with cardiovascular fitness, frailty, fall risk, and other health-related outcomes are generally low to moderate (i.e., ranging from 0.46 to 0.54). Nonetheless, a strong correlation (r = 0.71) has been identified between the 2MST and the Timed Up and Go performance, suggesting that higher step counts are associated with better mobility and lower functional limitations [3]. The relatively low capacity of the 2MST to identify health outcomes may be related to the test typically only counting the number of step repetitions completed, which ignores aspects of how the test is performed. While step count is a valuable indicator of gross motor output, it fails to capture critical characteristics such as cadence, cycle time, rate of performance change, and performance variability. These additional features could be instrumental in identifying subtle mobility deficits, early signs of frailty, and elevated fall risk [4,5].

Identifying additional movement features of the 2MST would usually require advanced instrumentation, such as force platforms and/or video- or inertial-sensor-based motion capture systems, which, despite their accuracy, are costly, complex, and in some cases, not portable. In addition, depending on the type (e.g., infrared, RGBD) and number of cameras in the motion capture system, intermittent occlusion may occur, resulting in occasional data loss. Motion capture system software routinely uses gap-filling routines to overcome such issues, which are problematic only when the gap becomes large (e.g., greater than 10 frames). Although occlusion remains a general limitation of video-based motion-capture approaches, the present protocol was designed a priori to minimize occlusion risk (i.e., a simple thigh-focused setup and limited arm swing), thereby reducing the likelihood of data loss.

The need for such high-performance equipment may have hindered the wider use of 2MST in clinical applications [6,7]. Recent advancements in mobile technology have enabled the integration of accelerometer and gyroscope sensors into everyday smartphones, providing a low-cost, portable, and accessible alternative for analyzing complex movements [8,9]. Prior instrumented implementations of the 2MST/step-in-place task are relatively scarce and are most often reported within broader Senior Fitness Test batteries, predominantly using low-cost video cameras (e.g., Kinect) and, less frequently, hybrid RGBD + IMU setups. Smartphone-based solutions have also been proposed, but they typically focus on test scores (i.e., complete cycle count) rather than validating 2MST kinematic estimates against marker-based optical motion capture. Consequently, a direct comparison of analytical (AA) versus machine-learning (ML) processing of thigh-mounted smartphone IMU signals against a multi-camera motion-capture ground truth during the 2MST remains largely unaddressed. These embedded sensors can capture kinematic data with increasing precision, providing quantitative measurements of several movement features. This makes smartphones accessible tools for assessing functional capacity and health outcomes in low-cost, low-resource clinical settings. Measures derived from the smartphone were compared with those obtained using a multi-camera system because, until relatively recently, multi-camera systems have been the primary approach for determining segmental kinematics, and their accuracy and reliability are well established.

If proven accurate, the use of smartphones could democratize access to biomechanical and/or movement assessments by reducing reliance on laboratory-based systems [10,11], while providing multiple outcome variables needed for complex, robust predictive models of mobility deficits, frailty, and falls risk. Beyond replacing laboratory-based systems, smartphone/IMU approaches may also enable remote monitoring during rehabilitation (e.g., occupational therapy) and post-discharge follow-up, where scalable deployment and longitudinal tracking can be prioritized even if accuracy is modestly reduced, provided that standardized instructions and signal-quality control procedures are in place. Having high-quality, reliable data forms the foundation for meaningful analysis and interpretation. However, the raw sensor signals from a smartphone commonly contain noise, artifacts, and drift that, unless corrected, can cause problems when using a smartphone to assess the movement patterns of the person holding/carrying the phone. Converting the raw sensor signals into clinically useful metrics often requires extensive signal processing. In general, such procedures include filtering, normalization, segmentation, and numerical integration or differentiation to enhance signal fidelity and ensure precise event detection. Traditionally, signal-processing techniques (i.e., analytical techniques) require a complex data-processing pipeline, including resampling, drift correction, filtering, and signal integration, to obtain metrics that can be trusted for clinical decision-making, research conclusions, or performance evaluation. Analytical approaches (AA) are inherently sensitive to noise or motion artifact in the recorded signal, often rely on information from a single sensor axis, assume linear relationships between the measured signal and the underlying biomechanical quantity, and have limited capacity to accommodate large within- and between-participant variability. In contrast, machine-learning (ML) approaches use the recorded data to learn complex, often non-linear mappings between the recorded signals and the biomechanical output parameter. In essence, the approach finds the hidden patterns in the data without having to be explicitly programmed for every rule. In this context, ML techniques have emerged as powerful tools for automating and optimizing signal processing of wearable-sensor data [12]. By leveraging information across multiple axes and features, learning complex mappings, and accommodating individual differences, ML pipelines can identify complex patterns in noisy signals, reduce reliance on extensive preprocessing, and directly estimate clinically meaningful outcomes, often with accuracy comparable to or exceeding that of traditional analytical methods [13]. Importantly, ML approaches have been shown to improve the estimation of high-magnitude angular velocity peaks, which occur at a magnitude range that analytical methods are particularly susceptible to movement- and filtering-related artifacts and peak-detection errors [14]. So far, no study has directly compared analytical and ML approaches for processing smartphone data to extract key performance metrics for the 2-Minute Step Test (2MST). The aim of the present study was to demonstrate how a smartphone held to the thigh can be used to assess thigh kinematics to provide outcome measures of the 2MST, and whether a ML data processing approach produces kinematic variables showing better agreement with those determined from a multi-camera motion system (ground truth measure) compared to those determined using a typical analytical data processing approach.

## 2. Materials and Methods

### 2.1. Participants

A convenience sample of 84 healthy young adults (1.71 ± 8.7 m; 66.9 ± 10.5 kg; and 28.7 ± 9.7 years) was recruited through the community of university students. Inclusion criteria were the ability to walk independently, no neurological disorders, no recent history of musculoskeletal disorders, and no ongoing pain during data collection.

### 2.2. Experimental Protocol

Participants performed the 2MST by marching in place for two minutes, lifting their knees to a target height, determined when standing, that was midway between the anterior superior iliac spine (ASIS) and the midpoint of the patella, as per standard protocols [1]. The height to which the lower limbs were raised was controlled by using a 1 m elastic band positioned horizontally directly in front of the participants, requiring them to touch the elastic band with the distal anterior aspect of their thighs at each step. After a short demonstration, a brief familiarization period was conducted before the experiment was undertaken.

### 2.3. Smartphone Data Collection

A smartphone (iPhone 12, Apple Inc., model A2172, Cupertino, CA, USA) was held to the anterior aspect at the top of the right thigh by the participant’s right hand, with the smartphone oriented vertically with the screen facing the participant’s body. The left hand (without phone) was held to the left thigh at the same height as the right hand was held to the right thigh. The angular velocity of the thigh segment was determined from the phone’s gyroscope sensor recorded at 60 Hz, using commercial software (SensorLog, version 6.1b).

### 2.4. Motion-Capture Data Collection

Simultaneously, thigh motion was recorded using a motion-capture system (Vicon, Oxford Metrics plc, Oxford, UK) comprising 11 infrared cameras, which tracked 2 reflective markers positioned over the greater trochanter (hip) and the protuberance of the lateral condyle (knee) of the right thigh. All procedures followed the manufacturer’s instructions and were similar to other studies that used motion analysis systems. The data were collected at a sampling frequency of 60 Hz to match the smartphone acquisition rate. The marker coordinate data were filtered using a 2nd order low-pass filter with a cut-off frequency of 6 Hz (value chosen based on residual analysis [12]). The hip and knee markers were used to define a 2D model of the right segment. The angle of the thigh segment was determined with respect to the vertical, with zero angle indicating the thigh was vertical, and 90° indicating the thigh was horizontal. The angular velocity of the thigh segment was determined as the first derivative of the thigh’s angular displacement. The data obtained from the motion capture system were considered a ground truth measurement of the thigh’s kinematics.

## 3. Signal Processing

### 3.1. Smart-Phone Analysis Approach

AA and ML data processing approaches for the phone’s angular velocity data were performed after preliminary data treatment steps. These preliminary procedures included signal adjustments, such as sampling rate and drift correction [13]. This was undertaken because small timestamp inconsistencies were observed in the raw smartphone data, indicating a non-constant sampling frequency (ranging from 58 to 62 Hz) and, in some instances, two samples shared the same timestamp. To correct these issues, the time-series data were resampled to a uniform 60 Hz sampling frequency using cubic spline interpolation. This procedure ensured that the gyroscope data from the smartphone consistently aligned temporally with the motion-analysis system’s data. Additionally, the smartphone sensor’s coordinate convention was adjusted so that upward and downward movements were represented as positive and negative values, respectively, matching the motion capture system’s convention.

**Figure 1:**
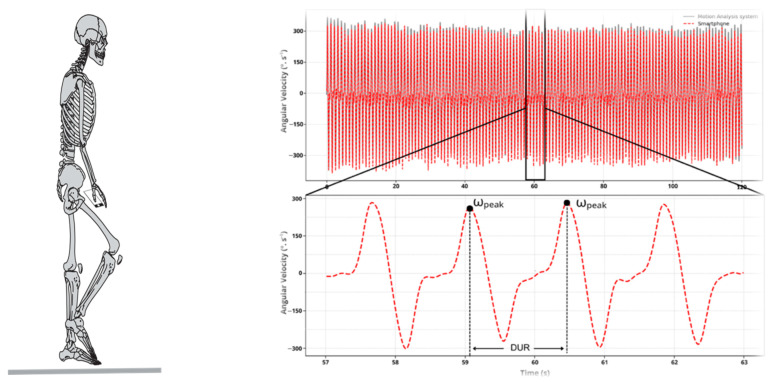
Schematic representation of the 2MST (**left panel**) and the temporal series (**upper right panel**) obtained from the smartphone (red dashed line) and the motion capture system (gray solid line), as well as a representation of the key instants of the cycle for the smartphone and motion-capture system (**lower right panel**).

**Figure 2:**
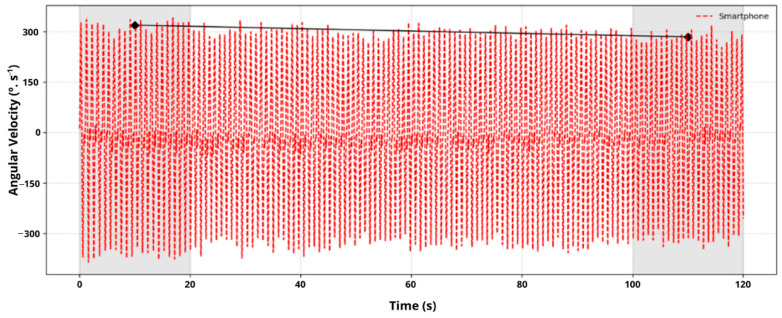
Exemplar of smartphone angular velocity data (red dashed line) from the 2MST depicting how RPC (solid black line) was determined. The RPC line is drawn between the mean angular velocity value of the initial 20 s of the test and the mean angular velocity value for the final 20 s of the test (shaded areas). In this sampler trial, the descending line indicates that the RPC was descending.

After applying these preliminary data treatment procedures, the angular velocity data series obtained from the smartphone were used to calculate the magnitude (ω_peak_) and instant of the peak thigh angular velocity for each step cycle using both the AA and ML data processing approaches (details of each approach are provided below). The following variables were identified based on detecting the ω_peak_ for each step cycle and the instants they occurred: (a) the number of step cycles (STP)—determined by the number of ω_peak_ detected; (b) the cycle duration (DUR)—calculated as the time between successive ω_peak_. These variables are represented in Figure 1. In addition, the rate of performance change (RPC) was calculated for each participant by determining how the ω_peak_ changed over the 2 min of the test. This was achieved by determining the slope of the line connecting the mean velocity value for the initial 20 s of the test and the mean velocity value for the final 20 s of the test (Figure 2). Lastly, the coefficients of variability (across cycles) in the ω_peak_ (CVω_peak_) and in the DUR (CV_DUR_) were also computed.

From the RPC value determined, each participant’s performance over the two minutes was classified as either “steady” (i.e., they sustained minimal performance variation; range of ±11.5°·s^−1^), “ascending” (i.e., they increased their performance; >11.5°·s^−1^ or “descending” (i.e., they decreased their performance; >11.5°·s^−1^) [14]. The cutoff of 11.5°·s^−1^ was defined arbitrarily, and no inferential statistical tests were performed on RPC values because the small sample sizes in each of the resulting subgroups (steady, ascending, descending) precluded meaningful comparisons. Figure 2 presents the smartphone angular velocity data from the 2MST for one participant and indicates that the RPC for this participant was ‘descending’ (i.e., with a negative slope).

### 3.2. Analytical Approach (AA) to Data Processing

The smartphone gyroscope data (*x*-axis; indicating thigh flexion–extension) were filtered using an adaptive, second-order Butterworth low-pass filter. The cutoff frequency was continually updated over time based on the dominant cycle frequency of the gyroscope signal at a particular point in time. To implement this adaptation, the gyroscope signal was processed using a 10 s moving window with a 1 s overlapping window. Within each window, the signal was mean-centered, and a real-valued FFT was computed. The zero frequency component (0 Hz) was excluded, and the frequency associated with the maximum spectral magnitude was taken as the window’s dominant cycle frequency. The cutoff frequency for each window was obtained from a predefined linear mapping between the dominant cycle frequency and the cutoff frequency. This mapping was established using a data-driven RMS criterion [14]. Specifically, within a candidate range of cutoff frequencies (1–12 Hz, evaluated on a fine grid), the gyroscope signal was filtered repeatedly, and the RMS error relative to the motion-capture reference angular-velocity signal was computed for the same window. The cutoff yielding the minimum RMS error was treated as the optimal cutoff for that window. A linear model was then fitted to relate the window’s dominant cycle frequency to its corresponding optimal cutoff frequency, yielding the mapping used during subsequent processing.

After determining this mapping, step cycles were partitioned into an independent training set (70%) and a separate test set (30%) to enable a like-for-like comparison with the ML approach. The mapping parameters were estimated using only the training subset (where the motion-capture reference was available for the RMS-based selection). The finalized mapping was then fixed and applied to the test subset to perform dynamic (window-wise) filtering using only the smartphone signal (i.e., without using the motion-capture reference during filtering). Filtering was applied piecewise (window-by-window) using each window’s cutoff frequency, and overlapping filtered segments were merged in the overlap region to produce a single continuous filtered signal. Then, smartphone and motion-capture signals were time-synchronized using cross-correlation. The angular velocity peak (ω_peak_) for each step cycle was extracted using the ‘find_peaks’ function. Additional variables (STP, DUR, RPC, CVω_peak_, CV_DUR_) were subsequently calculated. All signal-processing steps and variable extractions were performed using a custom Python (version 3.12.5) routine.

### 3.3. Machine Learning (ML) Approach to Data Processing

The thigh peak angular velocity measured by the motion-capture system was used as the supervised learning target (ground truth). Smartphone tri-axial gyroscope signals (x, y, and z axes) were filtered using a zero-lag, second-order low-pass Butterworth filter with a 6 Hz cutoff frequency [12]. Peak events were identified in the motion-capture reference angular-velocity signal, and fixed-length analysis windows (±0.12 s) were extracted from each smartphone gyroscope axis, centered at the time instant of each reference peak (the reference peak, detected on the *x*-axis, was used as the temporal anchor for the other axes). For each window and each smartphone axis, a set of time-domain predictors was computed, including peak value, selected percentiles, mean, standard deviation, root mean square (RMS), signal energy, high-amplitude width, number of zero crossings, and cycle duration. Additionally, the parabolically interpolated peak angular velocity of the smartphone signal in each axis was included as a predictor.

The ML model consisted of a stacked regression framework [15] combining two base learners: (1) a linear ElasticNet model [16] and (2) a histogram-based gradient boosting regressor [17,18]. The ElasticNet base learner (ElasticNetCV) was implemented within a preprocessing pipeline including median imputation, feature standardization, and variance filtering, and was trained with combined L1/L2 penalties to handle correlated predictors and reduce overfitting [16]. The nonlinear component was a histogram-based gradient boosting regression tree model (HistGradientBoostingRegressor), which can capture nonlinear relationships and interactions between predictors [17,18]. Stacking was implemented by generating out-of-fold predictions from each base learner under subject-wise cross-validation and fitting a Ridge regression meta-learner (RidgeCV) on these out-of-fold predictions [15,19]. All models and routines were implemented in Python using scikit-learn [20]. The stacked predictions were then calibrated using isotonic regression (with out-of-bounds clipping) implemented in scikit-learn [20]. Finally, to improve accuracy specifically for defining high-magnitude peaks, an additional linear correction was fitted to the upper tail of the calibrated predictions (top 5%) and applied only above the corresponding prediction threshold.

To evaluate generalization for unseen data (i.e., data from new individuals), a two-stage data partitioning and validation strategy was used, combining an independent train–test split with subject-wise cross-validation. First, all step cycles were randomly partitioned into two independent subsets: a training set comprising 70% of all cycles (6912 cycles) and a separate test set comprising the remaining 30% of the cycles (2962 cycles). The test set was not used during model fitting, hyperparameter tuning, stacking, calibration, or model selection. Within the training set, subject-wise cross-validation was applied using leave-one-subject-out (LOSO) logic via grouped splitting (e.g., GroupKFold), ensuring subject-level independence and reducing overfitting. At each fold, all cycles from participants selected for training purposes were included in the validation set, while the base learners were trained on the data from the remaining participants. Out-of-fold predictions from each base learner were then used to train the Ridge meta-learner, ensuring that the meta-model was fitted only on predictions generated from data not seen by the corresponding base learner during that fold [15]. Subsequently, the final stacked model was refitted using all data from the training subset and then evaluated on the test set, yielding an application-relevant estimate of generalization. The same variables calculated in the AA were then computed from the ML-derived outputs using a custom routine (Python, version 3.12.5).

### 3.4. Statistics

To evaluate whether smartphone-based inertial measurements can provide an accurate assessment of the 2MST, a Bland–Altman [21] analysis was conducted to assess agreement between the ω_peak_ values derived using the smartphone AA and ML approaches and those determined from the motion capture system. The agreement, including 95% limits of agreement, indicated the ± systematic offset (bias) between each smartphone approach (AA, ML) and the motion capture system (i.e., the ground truth). Homoscedasticity was assessed by visually inspecting whether the dispersion of agreement values remained constant across the range of measurement means. If a non-uniform pattern was observed, proportional bias was assessed by fitting a regression line between the agreement values and means. The Bland–Altman plots were performed only for the ω_peak_ and the cycle duration, as other variables (e.g., cadence) are calculated from these parameters. In addition, the coefficient of variability (CV) was determined for ω_peak_ and DUR (i.e., CVω_peak_ and CV_DUR_). All analyses were conducted in Python (version 3.12.5).

## 4. Results

All participants completed the 2MST without reporting any difficulty, discomfort, or pain during testing. The number of step cycles used (n = 2962) to determine agreement (unseen data) corresponded to 30% of the total sample. The average duration of these repeated cycles was identical across the different measurement modalities (motion capture system and smartphone), and across the different smartphone data-processing pipelines (i.e., AA or ML). On average, participants performed 143 ± 18 cycles (range from 111 to 185 cycles), with a duration of 0.84 ± 0.11 s (range from 0.65 to 1.06 s). The mean cadence across participants determined using the motion capture system was 72 ± 9 cycles·min^−1^ (range from 56 to 92 cycles·min^−1^). Since the number of cycles and the duration were identical between measurement modalities, no further comparisons were performed for cadence. The mean coefficient of variation in cycle duration was 3.9 ± 1.6% (range from 2.1 to 9.7%). The mean peak thigh angular velocity (ω_peak_) obtained from the motion capture system was 303 ± 39°·s^−1^ (range from 204 to 444°·s^−1^). Compared to the motion capture system (ground truth), the mean ω_peak_ obtained using the AA was underestimated by −7.5% (280 ± 47°·s^−1^; range from 150 to 433°·s^−1^), while the mean ω_peak_ obtained using the ML approach was nearly coincident (+0.3%; 304 ± 37°·s^−1^; range from 198 to 432°·s^−1^).

**Figure 3:**
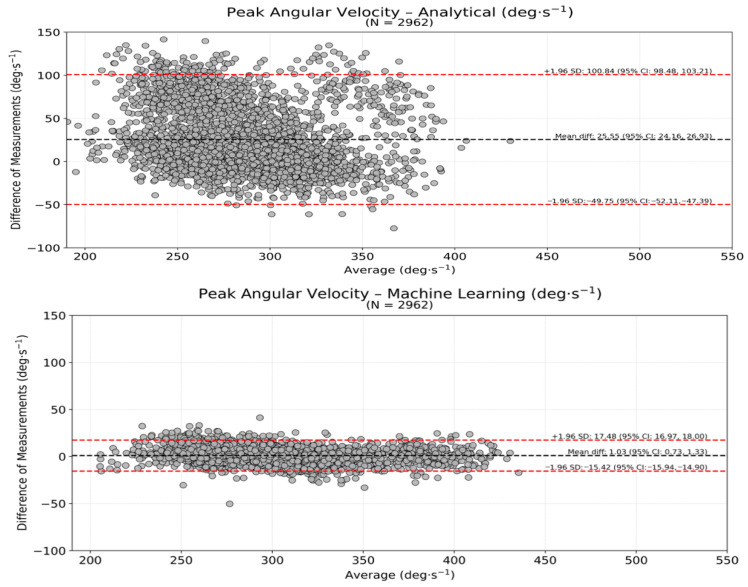
Bland–Altman plots for the thigh peak angular velocity (ω_peak_) showing agreement between the motion capture system-derived approach and the AA (**upper panel**) and the ML (**lower panel**) smartphone-derived approaches (2962 cycles). The red-traced lines represent the 95% upper and lower limits of agreement, which are also numerically indicated.

The Bland–Altman agreement analysis indicated that the ω_peak_ estimated from the AA presented a bias of 25.5°·s^−1^, with limits of agreement of −49.8–100.8°·s^−1^ (Figure 3—upper panel) with respect to the outcomes identified by the motion capture system. The agreement for the ω_peak_ of the ML approach showed a bias of 1.0°·s^−1^, with limits of agreement of −15.4–17.5°·s^−1^ (Figure 3, lower panel). Figure 3 presents the ω_peak_ agreement derived from the smartphone using either the AA (upper panel) or the ML (lower panel) approaches with that determined by the motion capture system.

**Figure 4:**
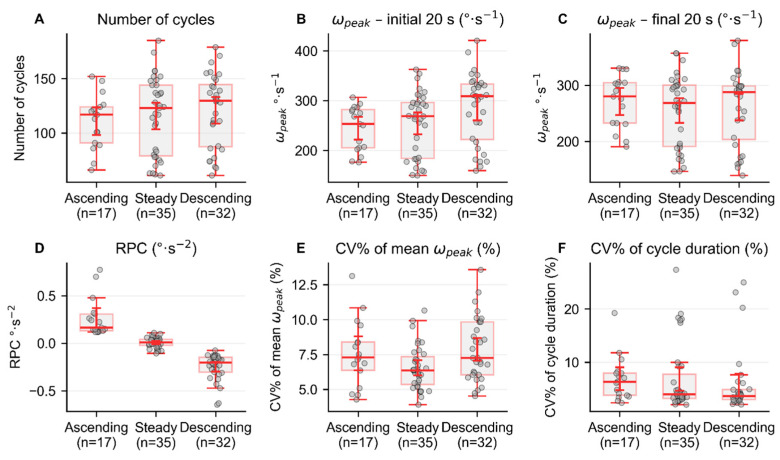
Box and whisker plots of the mean number of cycles (**A**), the mean ω_peak_ for the initial (**B**) and the final 20 s (**C**) of the 2MST, the RPC (**D**), and the mean coefficient of variability of the ω_peak_ (CVω_peak_) (**E**) and cycle duration (CV_DUR_) (**F**) for the three sub-groups: steady, ascending, and descending strategies.

The analysis of the rate of performance change (RPC) indicated that the largest proportion of participants (n = 35; ~41.6%) maintained a relatively constant (i.e., steady) performance throughout the test. Approximately 38.1% of participants decreased their performance by 6.1% (descending), and 20% increased their performance by 10.8% (ascending). Figure 4 indicates, for the three sub-groups (steady, ascending, and descending strategies), the mean number of cycles, the mean ω_peak_ values for the initial and final 20 s of the 2MST, the RPC, and the mean coefficient of variability of ω_peak_ and DUR.

## 5. Discussion

The present study shows that positioning a smartphone on the thigh can capture lower-limb kinematics during the 2MST using the device’s embedded inertial sensors. The study is comparable with prior work that used thigh-mounted smartphones to quantify stepping and dynamic balance tasks [22,23]. The approach goes beyond counting step repetitions by quantifying other 2MST parameters (i.e., peak thigh velocity, cycle duration, cadence, rate of performance change, and variability). Quantifying additional parameters may help improve the predictive ability of using the 2MST to evaluate functioning capacity/health outcomes (e.g., frailty, falls, and other clinical outcomes). The study also found that a ML data processing approach yielded data nearly identical to ground truth motion capture data, whereas data processed using a typical analytical approach showed errors of around 8%.

### 5.1. Number of Cycles

An excellent agreement was observed between the cycle count obtained with the smartphone and that derived from the motion capture system, confirming the reproducibility and data processing automation proposed in the present study. The agreement in the number of cycles was reached irrespective of the data-processing pipeline (i.e., AA vs. ML). The cycle count was determined by detecting the repeated, obvious, and distinct positive peaks in the thigh angular velocity. This is an easily observable event-based parameter that is readily detectable even in minimally processed data. Therefore, as long as the sensor signal preserves the general pattern of the rising and lowering of the limbs when stepping in place, the total number of cycles remains stable across different analytical approaches.

Accurate repetition counting is often the primary outcome of many functional assessments, including the 2MST. Recent studies have shown that observer counting is susceptible to inter- and intra-rater errors, even when using 30 s assessment protocols [24,25], and to mitigate such errors, two evaluators have been recommended [25]. In the 2MST, the likelihood of discrepancies is higher because of the longer duration of the test (i.e., 120 s). Indeed, it has been reported that when determining repetition number, there is a mean difference of 3–5 cycles between in-person observation and video analysis, and in some cases, discrepancies may be larger [26].

Studies that have administered the 2MST in healthy participants reported an average of 110 cycles in older adults [1] and 120 cycles in middle-aged adults [27]. These cycle numbers are consistent with the mean cycle count identified in the present study. Despite the similar mean number of repetitions, there was considerable inter-subject variation (79–144 cycles). The wide range of performances may have resulted from participant differences in physical capacity/endurance or in their interpretation of the task. Irrespective of interindividual variations, the smartphone-based inertial measurement approach (both AA and ML) counted the same number of cycles as the motion capture system. This suggests that the approach could be used to assess clinical populations that are likely to exhibit a wide range of performance variation.

### 5.2. Cycle Duration/Cadence

The present study showed excellent agreement in cycle duration obtained with the smartphone and that derived from the motion capture system (mean bias = 0.0005 s; CI 95%: 0.0035–0.0026 s), resulting in identical cadence detection between the measurement systems. Cycle duration and cadence are fundamental descriptors of gait that have been used to indicate shifts in the temporal aspects of movement coordination and control, which are mirrored by biomechanical and physiological adaptations. The mean cadence of 55 cycles·min^−1^ is comparable to that reported in other studies that used a similar protocol (51–60 cycles·min^−1^ [28]). It should be emphasized that cadence is not usually controlled during test administration [1] and was not controlled in the current study. Previous research indicates that increases in cadence increase metabolic cost [29], and atypical cadence or cycle duration (i.e., increased step cycle variation) is associated with poorer balance performance and a greater risk of falls in older adults [30]. Although the cadence in the current study was comparable to previous reports, there was substantial variation in cycle duration across participants. Again, this may be related to differences in participants’ physical capacity/endurance, or to their interpretation of the task (as highlighted above).

### 5.3. Peak Angular Velocity (ω_peak_)

The agreement between smartphone-derived and motion capture system measurement was excellent for angular velocity outcomes, particularly for the ML data processing approach. The ML pipeline yielded a very low systematic bias for determining peak angular velocity (1.0°·s^−1^) relative to the motion capture approach (ground truth), and substantially narrower limits of agreement (±16.4°·s^−1^) when compared to the analytical data processing approach (25.5°·s^−1^ ± 50°·s^−1^). These findings suggest that the ML approach was effective at capturing the magnitude of angular velocity peaks across cycles, with high consistency. In contrast, the analytical data processing approach showed poorer agreement, with limits of agreement approximately 4.5 times larger than those obtained with the ML approach. This substantial discrepancy highlights that traditional analytical pipelines may be sensitive to signal-processing choices, such as the filtering used and the timing and method of signal differentiation. The markedly better agreement and narrower limits observed with the ML approach indicate superior robustness to these error sources, likely because the ML approach learned systematic relationships between the raw smartphone signal and the ground-truth reference signal. Collectively, these results underscore the potential advantages of ML-based methods for improving the precision and reliability of angular velocity estimation in field-based assessments, supporting its use as an alternative to conventional analytical pipelines.

Unlike monitoring repetition count, determining changes in the peak angular velocity across cycles provides insight into the quality of movement execution rather than merely its quantity or timing. Assessing the angular velocity peaks reflects the performer’s ability to generate rapid joint motion during the concentric phase of movement, which is closely linked to neuromuscular power production at the hip and ankle joints [31,32]. In the context of the 2MST, this parameter captures how effectively the hip flexors and plantarflexor muscles move and lift the lower limbs to achieve the required limb height at each step, thereby integrating assessment of strength, coordination, and neural drive into a single performance metric. As such, assessing the angular velocity peaks provides a better representation of the biomechanical and neuromuscular demands of the task than temporal or count-based outcomes alone.

Importantly, velocity measures have been shown to be more sensitive indicators of functional capacity and decline than maximal strength assessments, particularly in older adults and clinical populations. As highlighted by Reid and Fielding [33], reductions in muscle power occur earlier and progress more rapidly with aging than losses in maximal force, making power-related metrics more informative for detecting early functional impairments. In this regard, assessing the thigh angular velocity peaks may serve as a valuable proxy for changes in lower-limb power during the 2MST, enabling the identification of subtle performance deficits or compensatory strategies that are not evident from repetition count or cadence. Consequently, incorporating thigh angular velocity peak measures into the assessment framework may enhance the 2MST’s sensitivity for detecting declines in functional performance and for characterizing the movement strategies adopted to successfully complete the test. However, the ability to assess thigh angular velocity peak during the 2MST to predict cardiovascular fitness remains to be established.

In the present study, the ML approach showed superior agreement with motion-capture–derived thigh peak angular velocity compared with the analytical signal-processing pipeline. This finding is consistent with recent evidence indicating that data-driven models can better capture the complex relationship between inertial sensor signals and true segmental kinematics than rule-based analytical methods, particularly under variable movement conditions [34,35]. Analytical approaches typically rely on predefined filtering and peak-detection rules applied directly to the smartphone gyroscope signal, implicitly assuming a stable and largely linear correspondence between local signal features and underlying segmental motion. While computationally efficient and transparent, such methods are sensitive to noise, axis misalignment, and inter-individual variability in movement strategies—limitations that are well documented in wearable-sensor–based biomechanical assessments [34,36]. Similar advantages of ML regression models over AA pipelines have been reported for estimating joint kinematics and spatiotemporal gait parameters from inertial sensors in both laboratory and real-world settings [35,37]. These issues are especially relevant in functional stepping tasks, where fatigue and compensatory strategies may alter signal morphology across cycles/time. By training the model using motion capture as the ground truth, the present pipeline accounted for axis cross-talk, nonlinear relationships, and interactions among time-domain features derived from all three gyroscope axes. ML-based approaches explicitly learn the empirical mapping from multi-axis inertial sensor features to a reference standard, which is crucial for mitigating inter-axis crosstalk arising from sensor misalignment relative to anatomical axes and from soft-tissue artifact. AAs typically try to mitigate such issues by selecting a single “dominant” axis or by computing a vector magnitude, thereby implicitly assuming a stable, orthogonal relationship between sensor and anatomical frames. Such assumptions are rarely satisfied in free-living or functional tasks and may vary across participants and cycles. On the other hand, ML incorporates information from all three gyroscope axes simultaneously and allows the model to learn how these axes jointly contribute to the ground-truth thigh angular velocity. By using multi-axis time-domain features as predictors, the stacked regression framework implicitly learns axis weighting and cross-axis compensation, rather than imposing a priori assumptions about axis relevance. As a result, inter-axis crosstalk is treated as a learnable property of the data, enabling the ML model to accommodate sensor-to-segment misalignment and axis coupling in a data-driven manner.

The relationship between smartphone gyroscope features and true segmental angular velocity is inherently nonlinear, particularly at higher movement speeds and when movement patterns deviate from stereotypical trajectories. Conventional AA pipelines based on linear filtering and peak-detection rules implicitly assume proportional scaling between the sensor signal and the underlying biomechanical quantity. When this assumption is violated, particularly across different velocity ranges, analytical methods are prone to systematic bias and reduced accuracy, most notably for high-magnitude angular-velocity peaks. To address these limitations, a stacked regression framework was implemented for the ML approach that combined a regularized linear model (ElasticNet) with a nonlinear histogram-based gradient-boosting regressor. The linear component captured stable, approximately linear relationships while mitigating multicollinearity and overfitting through L1 and L2 regularization, thereby preserving interpretability. In parallel, the nonlinear tree-based learner modeled higher-order interactions and nonlinear scaling effects across features and sensor axes. This hybrid architecture enables the ML model to adapt across different movement regimes and velocity ranges, improving generalization and robustness relative to purely analytical approaches. Consistent with prior biomechanical time-series research, such ensemble frameworks effectively capture both linear trends and complex nonlinear dynamics, reducing estimation bias and enhancing accuracy, particularly in ranges where AA methods are most susceptible to filtering artifacts and peak-detection errors. Notably, the present ML approach also improved accuracy for high-magnitude angular velocity peaks, which is a magnitude range that analytical methods are particularly susceptible to filtering artifacts and peak-detection errors [36]. Such hybrid architectures have been shown to improve generalization and reduce overfitting in biomechanical time-series prediction tasks by capturing both stable linear trends and more complex nonlinear effects [37,38].

Inter-individual differences in movement strategy—such as step/thigh height, cadence, trunk involvement, and compensatory patterns—substantially alter the morphology of gyroscope signals during the 2MST. AAs typically apply uniform signal-processing rules across all participants, thereby assuming homogeneous movement patterns and consistent signal morphology. By contrast, the present ML approach is trained directly against the motion-capture–derived peak angular velocity and uses a diverse feature set that summarizes both local peak behavior and cycle-level signal characteristics. The use of regularization, ensemble learning, and out-of-fold stacking enables the model to generalize across participants while still accommodating individual-specific signal patterns. Consequently, participant-specific movement strategies are reflected in the learned mapping between smartphone features and the reference signal, rather than being treated as noise or as violations of model assumptions.

Although ML models are sometimes viewed as less interpretable, the use of regularization, standardized features, and a linear component in the present pipeline supports transparency while maintaining predictive performance. Recent studies have emphasized that such balanced ML approaches are well-suited for clinical and field applications, where signal quality and movement patterns are inherently more variable than in controlled laboratory environments [34,35]. Collectively, these findings support the use of ML-based methods as a robust and scalable alternative to traditional analytical pipelines for estimating thigh angular velocity from smartphone sensors under real-world assessment conditions. Although the use of other devices and AAs may yield lower accuracy, this is not necessarily a problem when assessing dichotomous parameters (e.g., when determining positive or negative characteristics of a signal to ascertain step cycle count). However, when additional features of performance on the 2MST are required, e.g., an evaluation of the peaks in the thigh angular velocity to determine performance variability and/or indication of performance decrements (fatigue index). Then, the AA would be inappropriate; instead, the ML approach should be used. Thus, when to use an ML approach should be determined based on the context of what is being investigated. The present study emphasizes that ML provides a more accurate method, and this may be particularly relevant when predictive models are to be established using such data.

### 5.4. Rate of Performance Change (RPC)

The present study defined the rate of performance change (RPC) as either steady, ascending, or descending. Most participants sustained (41%) or reduced (38%) their performance over the two minutes of the test. Any declines in performance are likely linked to the participant’s ability to continuously activate the muscle groups involved in the task (i.e., neuromuscular fatigue [39]). It is also plausible that the declines in performance are linked to several factors, such as cardiovascular capacity [40], gender differences, age variation, and motivational drive [41], which are beyond the scope of the present study. The performance decline cannot be compared with findings from other studies, as such information is unavailable in previous work. Detecting the rate of performance change could be a promising approach when using the 2MST to assess aerobic endurance and lower-body functional fitness in clinical populations, as it may reveal the use of different strategies in response to differences in physiological capacity, motor control, and self-regulation. Future research is required to determine if the RPC is related to aerobic endurance and/or lower-body functional fitness.

### 5.5. Limitations

The present study has several limitations. First, the smartphone was held to the participants’ thigh with their right hand, which may have produced minor movement artifacts (e.g., movements of the underlying tissues, small displacements, rotations, or gradual drifts of the smartphone). Holding the phone to the thigh may constitute a challenge for very old or neurological participants, as they may struggle to sustain the device in place. On the other hand, holding the smartphone eliminates the burden of additional apparatus (satchels, Velcro straps, tapes, etc.) that if are required to be used may limit the ease and simplicity of the test application. Furthermore, the excellent agreement between the smartphone and true-ground measurements indicates a minimal influence of the way the smartphone was secured. Second, the current study assessed young, healthy participants, and did not control cadence. The lack of cadence control may explain the large inter-subject variation in step count and cycle variability. However, cadence is typically not controlled when administering the 2MST, and indeed, freely chosen cadence may constitute an important parameter for identifying relevant aspects of the movement. As cadence was not controlled, there was a wide range in the number of step counts completed across participants. This can be seen as a positive aspect as it meant comparisons between measurement modalities and/or data processing pipelines were made over a wide range of performances. The use of an arbitrary cutoff of 11.5°·s^−1^ was arbitrarily established and may be viewed as a limitation, as it may vary depending on participants’ characteristics (e.g., older adults, clinical patients). Although the number of repetitions completed and the cadence used by the young participants in the present study fall within the mean ranges reported for 2MST for older adults and patients with clinical conditions, further work is required to determine the validity of the presented approach for the evaluation of data collected from older adults or clinical populations. It is worth noting that different cutoffs applied to identify strategies (e.g., ascending, steady, or descending) may produce distinct classifications. Third, only one smartphone brand/model was used in the current study; thus, it is not known to what extent the results can be generalized to other smartphone models and brands due to differences in sensor specifications. Finally, readers should be aware that alternative analytical and machine learning data-processing pipelines may produce different outcomes and influence comparisons between approaches.

## 6. Conclusions

This study demonstrated that kinematic parameters derived from a smartphone held against the thigh provided a valid assessment of the 2-Minute Step Test (2MST) by showing excellent agreement with comparable measurements from a motion capture system. Step cycle count and cycle duration demonstrated high consistency between measurement modalities (smartphone, motion capture system) despite relatively large inter-subject pacing differences. The use of the ML data processing approach to process the phone’s sensor signal yielded more accurate data than the data produced using a typical analytical data processing approach. That is, when using the ML data processing approach, smartphone-derived peak angular velocity measures showed strong agreement with the ground truth measure, with a mean discrepancy of only 1.0°·s^−1^, whereas when using the AA data processing approach, the discrepancy was 25.5°·s^−1^. These results suggest that smartphone sensors can detect meaningful performance metrics and support the validity of smartphone-based assessment of the 2MST. The ability to extract multiple parameters with accessible mobile technology enables the 2MST to serve as a scalable, low-cost tool for remote or clinical functional assessments. Further research is needed to determine the prognostic value of these sensor-based metrics in predicting health outcomes across diverse patient groups.

## Data Availability

The data presented in the study are openly available in https://doi.org/10.17632/b6mn8swzwm.1.
